# The Effect of Yupingfeng Polysaccharides on Immune Performance and Intestinal Microbiota in Goslings

**DOI:** 10.3390/ani15142077

**Published:** 2025-07-14

**Authors:** Qinxin He, Miaoxin Huang, Tianyu Wang, Li Gong, Zheng Ma, Fei Ye, Hua Li

**Affiliations:** Guangdong Provincial Key Laboratory of Animal Molecular Design and Precise Breeding, School of Animal Science and Technology, Foshan University, Foshan 528225, China; 18244408868@163.com (Q.H.); 13432640730@163.com (T.W.); 13690192358@163.com (M.H.); gongli2018by@zju.edu.cn (L.G.); mz8522@163.com (Z.M.); yefei0831@fosu.edu.cn (F.Y.)

**Keywords:** Yupingfeng polysaccharides, gosling, microflora, immune, intestinal health

## Abstract

This study evaluated the effects of Yupingfeng polysaccharides (YPF-P) on production performance, immune response, and intestinal health in goslings. A total of 240 one-day-old healthy male goslings were randomly assigned to four groups, each with six replicates of ten goslings. The Control group (Control) was fed a basal diet, while the experimental groups received the basal diet supplemented with 200 mg/kg (YPFPI), 400 mg/kg (YPFPII), and 600 mg/kg (YPFPIII) of YPF-P. The results demonstrated that supplementation with 400 mg/kg of YPF-P significantly decreased the final body weight at 21 days and the feed conversion ratio (FCR) from days 1 to 14 (*p* < 0.05). Plasma activities of glutathione peroxidase (GSH-Px) and superoxide dismutase (SOD) were significantly elevated, while malondialdehyde (MDA) levels were reduced in the 400 and 600 mg/kg groups (*p* < 0.05). Both dosages significantly increased thymus and bursa of Fabricius indices, as well as plasma IL-1β concentration (*p* < 0.05), with IL-6 levels further elevated in the 600 mg/kg group (*p* < 0.05). Duodenal and ileal villus height and the villus height to crypt depth ratio were significantly improved in the 400 and 600 mg/kg groups (*p* < 0.05). In the cecum, acetate and isobutyrate concentrations were increased in the 400 and 600 mg/kg groups, while propionate concentration was significantly higher in the 600 mg/kg group (*p* < 0.05). The 600 mg/kg group also exhibited a significant increase in the relative abundance of beneficial bacteria such as *Akkermansia* and *Alistipes*, alongside a marked reduction in harmful pathogens, including *Rickettsia* (*p* < 0.05). In summary, dietary supplementation with YPF-P enhanced antioxidant capacity, immune response, and gut microbiota composition in goslings, with the most pronounced effects observed at 600 mg/kg.

## Simple Summary

The prudent use of antibiotics is vital for safeguarding both animal and human health. As a natural and safe alternative, Chinese herbal medicines offer promising potential, as they contain no synthetic drug residues that could remain in animal tissues. Compared to single-herb applications, well-formulated combinations of multiple herbs can exert synergistic effects, enhancing their therapeutic efficacy while also contributing to better economic outcomes. Yupingfeng polysaccharide was prepared by weighing powdered crude drugs of *Astragalus membranaceus* (Huang qi), *Atractylodes macrocephala* (Bai zhu), and *Saposhnikovia divaricata* (Fang feng) in a weight ratio of 3:1:1. The results showed that Yupingfeng polysaccharides were found to improve gut microbiota balance and intestinal health in goslings while potentially lowering antibiotic residue risks.

## 1. Introduction

Antibiotics have been widely used in livestock and poultry farming for disease prevention, treatment, and growth promotion, effectively improving animal health and production efficiency [1]. However, their misuse has led to issues such as antibiotic residues and antimicrobial resistance, posing threats to food safety and public health [2]. In reaction, the Chinese agricultural authority outlawed the inclusion of antibiotics in animal feed beginning in July 2020 [3]. The complete ban on antibiotics necessitates the search for alternative solutions to protect poultry health, making the development of antibiotic alternatives a hot research topic.

Recently, phytogenic substances have become part of the feed additive market, which are valued for their green and wholesome properties. They are widely applied in modern poultry production systems. Due to their strong biological effects, these compounds can boost immune function [4], reduce oxidative stress [5], repair gut barriers [6], and balance gut microbes [7], thereby enhancing growth performance and farm profitability. Yupingfeng powder (YPF), a classic herbal remedy containing *Astragalus membranaceus*, *Atractylodes macrocephala*, and *Saposhnikovia divaricata*, acts as a natural immune modulator, demonstrated with anti-inflammatory and anti-tumor properties. Its primary bioactive polysaccharide component, Yupingfeng polysaccharides (YPF-P), enhances growth performance by improving intestinal mucosal integrity and restoring gut microbiota balance while amplifying systemic immune regulation [8,9]. Hu et al. [10] reported that YPF-P strengthens piglets’ immune response through elevated production of classical swine fever antibodies and immunoglobulins. Zhou et al. [11] found that both YPF-P and YPF-P liposomes improved the histological structure of immune organs in chicks, significantly increased the number of Bu-1^+^ B lymphocytes and the CD4^+^/CD8^+^ T lymphocyte ratio, promoted cell proliferation and differentiation, and enhanced overall immunoregulatory capacity. In addition, studies have found that supplementation with YPF-P improved gut microbiota composition by increasing beneficial bacteria such as *Bacteroidetes*, *Proteobacteria*, *Veillonellaceae*, and *Muribaculaceae* in piglets, and *Faecalibacterium*, *Bacteroides*, and *Enterococcus* in poultry [12,13]. These modifications enhanced polysaccharide degradation, nutrient absorption, and gut health, ultimately promoting growth performance and reducing diarrhea incidence. Therefore, YPF-P can be utilized as a feed additive in livestock and poultry production. This approach not only effectively enhances production performance and improves disease resistance in animals, but also facilitates the recycling of medicinal herb residues. Importantly, extensive production practices have demonstrated that long-term supplementation with YPF-P does not result in antimicrobial resistance or residue accumulation in animals.

China currently holds a leading position in the global goose industry. Goslings are highly sensitive to environmental factors, and inadequate management can result in reduced immune function and high mortality rates, representing one of the major challenges in goose production. However, there are few reports on the use of YPF-P as an antibiotic substitute and other clinical studies in poultry [14]. This experiment investigates the effects of adding 200 mg/kg, 400 mg/kg, and 600 mg/kg of YPF-P to gosling feed on serum immunity and intestinal morphology. The cecal contents were subjected to 16S rRNA sequencing to detect changes in the composition and abundance of gut microbiota. The results establish a foundation for utilizing YPF-P as a sustainable and harmless dietary additive in avian production systems.

## 2. Materials and Methods

### 2.1. Materials

All animal experiments were conducted in strict accordance with the Animal Management Regulations issued by the Ministry of Health of the People’s Republic of China. Ethical approval was obtained from the Institutional Animal Care and Use Committee of Foshan University (approval no. FOSU 2023-167). Humane euthanasia was performed upon completion of this study. The gosling geese were purchased from Jiangmen Jiaqin Kang Aquaculture Technology Service Co., Ltd. (Jiangmen, China). The Yupingfeng polysaccharides, an alcoholized product of Yupingfeng, were provided by Sinopharm Group Dezhong Pharmaceutical Co., Ltd. (Foshan, China). The formulated feed for goslings was purchased from Qingyuan Jinzheng Feed Co., Ltd. (Qingyuan, China), and the feed formula was designed with reference to the goose nutritional requirements (0~3 weeks old) in NRC (1994). Details of the basal diet formulation and its nutrient composition are presented in Table 1.

### 2.2. Animal Treatments and Design

A total of 240 healthy male goslings with no significant differences in initial mean body weight (*p* > 0.05) were randomly allocated into 4 groups (6 replicates per group, 10 birds per replicate) for a 21-day feeding trial. The Control group was fed a basal diet without YPF-P supplementation (0 g/kg), whereas the treatment groups received the basal diet supplemented with 200, 400, or 600 mg/kg of YPF-P, respectively. The gosling geese were raised indoors in pens (81.5 cm × 36.5 cm × 32.8 cm) with the environment thoroughly sanitized before the experiment. The goose house temperature was maintained at 28–30 °C during the first week, then reduced by 1–2 °C weekly until reaching ambient temperature by the third week. The goslings were provided 24 h of continuous light for the first week, which was gradually reduced to 12 h/d by the second week and transitioned to natural lighting by the third week, with a light intensity of 10–20 lx. The humidity in the goose house was controlled at around 65 ± 5%. Manure was regularly disposed of to maintain ventilation and dryness. The geese had free access to water and food, with small meals and frequent feeding. Vaccinations were administered according to the routine immunization schedule. During the experiment, the goslings’ mental and health status was observed, and weak goslings were promptly eliminated.

### 2.3. Measurement of Growth Performance

To accurately determine the average daily gain (ADG), average daily feed intake (ADFI), and feed conversion ratio (FCR), body weight was measured at 08:00 a.m. on days 1, 7, 14, and 21 of the experiment. Feed was withheld for 6 h before weighing, but water was not restricted. The initial average body weight of the goslings on day 1 was recorded as the initial weight (IW), and the weekly average final weight was recorded as the weight at the end of the trial (FW). The growth status of the goslings was observed and recorded in real time during the trial. The feed consumption, feed intake, and leftover feed were recorded for each replicate group.

### 2.4. Immune Organ Indexes

On day 21 of this study, six goslings with comparable body weight and healthy condition were randomly selected from each group (one gosling per replicate, with a total of ten replicates per group). Approximately 5 mL of blood was drawn from the pterygoid vein into glass tubes without anticoagulants, followed by centrifugation at 3500 r/min for 15 min. The separated plasma was stored at −20 °C for further biochemical analysis. After blood collection, all selected goslings were humanely sacrificed by cervical exsanguination. The spleen, thymus, and bursa of Fabricius were subsequently dissected and weighed. The immune organ index was determined using the following equation:

Immune Organ Index (g/kg) = Organ Weight/Body Weight

### 2.5. Blood Biochemistry

As mentioned in Section 2.4, the centrifuged blood samples were utilized for biochemical assessments. The plasma concentrations of immunoglobulin A (IgA), immunoglobulin G (IgG), immunoglobulin M (IgM), interleukin-1β (IL-1β), interleukin-6 (IL-6), and tumor necrosis factor-α (TNF-α) were determined using commercial ELISA kits obtained from Shanghai Yuanju Biotechnology Co., Ltd. (Shanghai, China). Serum levels of total antioxidant capacity (T-AOC), superoxide dismutase (SOD), malondialdehyde (MDA), and glutathione peroxidase (GSH-Px) were analyzed using corresponding assay kits supplied by the Nanjing Jiancheng Bioengineering Institute (Nanjing, China).

### 2.6. Morphological Analysis of the Duodenum, Jejunum, and Ileum

Following euthanasia, 2 cm segments of the duodenum, jejunum, and ileum were excised and immediately fixed in 4% paraformaldehyde. The tissues were then dehydrated through a graded ethanol series, cleared, and embedded in paraffin. Using intestinal sectioning technology (Wuhan Servicebio Technology Co., Ltd., Wuhan, China), 4 μm-thick sections were cut with a rotary microtome (HistoCore BIOCUT MCT-001; Leica, Wetzlar Germany) and stained with hematoxylin–eosin (H-E). Morphological evaluation was performed by examining three non-consecutive sections per intestinal segment at 100× magnification (three random fields per section). Villus height (VH) and crypt depth (CD) were measured on five intact, well-oriented villi per section using image analysis software (ImageJ v2.0.0, NIH, Bethesda, MD, USA), followed by calculation of the VH/CD ratio.

### 2.7. 16S rRNA Sequencing and Analysis

After collection, cecal contents were portioned into sterile 1.5 mL microcentrifuge tubes (n = 5), labeled, and rapidly frozen in liquid nitrogen for 15 min. The samples were subsequently stored at −80 °C until further analysis. Genomic DNA was isolated from 0.5 g of each cecal content using the TGuide S96 Magnetic Bead Soil/Feces DNA Kit (Tiangen Biotech, Beijing, China) following the manufacturer’s protocol. DNA integrity and concentration were assessed by 1.8% agarose gel electrophoresis. DNA purity and yield were determined using a NanoDrop 2000 spectrophotometer (Thermo Scientific, Waltham, MA, USA). The V3–V4 hypervariable regions of the bacterial 16S rRNA gene were amplified using primer pair 338F (5′-ACTCCTACGGGAGGCAGCA-3′) and 806R (5′-GGACTACHVGGGTWTCTAAT-3′), with both primers containing unique Illumina adapter barcodes [15]. PCR reactions were performed in a 20 μL reaction system containing 5–50 ng template DNA, 0.3 μL of each primer (10 μM), 5 μL KOD FX Neo buffer, 2 μL dNTPs (2 mM each), 0.2 μL KOD FX Neo DNA polymerase (Toyobo, Osaka, Japan), and nuclease-free water [15]. The PCR program consisted of an initial denaturation at 95 °C for 5 min, 25 cycles of denaturation at 95 °C for 30 s, annealing at 50 °C for 30 s, and extension at 72 °C for 40 s followed by a final extension at 72 °C for 7 min [12]. PCR products were purified using the Omega DNA purification kit (Omega Bio-tek, Norcross, GA, USA) and quantified with a Qsep-400 Biofragment Analyzer (BiOptic, Taiwan). The purified products were normalized to equimolar concentrations, pooled, and subjected to paired-end sequencing (2 × 250 bp) on the Illumina NovaSeq 6000 platform (Illumina, San Diego, CA, USA) by Beijing Novogene Bioinformatics Technology Co., Ltd. (Beijing, China).

High-quality sequences sharing ≥ 97% similarity were clustered into operational taxonomic units (OTUs) using USEARCH (version 10.0). Taxonomic assignment of OTUs/ASVs was conducted using the Naive Bayes classifier implemented in QIIME2, referencing the SILVA database (release 138.1), with a confidence threshold set at 70%. Alpha diversity was assessed to evaluate the species richness and diversity within each sample using QIIME2. Beta diversity was analyzed through principal coordinate analysis (PCoA) to compare microbial community composition among samples. One-way ANOVA was employed to compare bacterial abundance and diversity among groups. Linear discriminant analysis (LDA) combined with effect size measurements (LEfSe) was used to identify taxa with significant differential abundance. Sequencing data were analyzed using the online platform BMKCloud Correlation analyses were performed between gut microbial communities at the genus level and immune-related as well as antioxidant parameters [15]. Spearman’s correlation coefficients were calculated using the online platform OmicStudio (https://www.omicstudio.cn, accessed on 27 September 2023). Heatmaps were generated to visualize the pairwise correlations between variables; * *p* < 0.05, ** *p* < 0.01.

### 2.8. Short-Chain Fatty Acid (SCFA) Quantification

Around 50 mg of cecal content per sample was accurately weighed into sterile 1.5 mL microcentrifuge tubes (n = 5). Subsequently, 1000 μL of extraction solvent containing internal standards (methanol, acetonitrile, and water mixed at a volumetric ratio of 2:2:1) was added and thoroughly vortexed for 30 s. Steel beads were added, and the mixture was homogenized using a bead mill for 10 min, followed by sonication in an ice water bath for 10 min and incubation at −20 °C for 1 h. The samples were subsequently centrifuged at 12,000 rpm at a low temperature for 15 min. A 500 μL aliquot of the resulting supernatant was transferred to a fresh centrifuge tube. The extract was vacuum-dried, reconstituted, and subjected to a second centrifugation. Finally, the supernatant was transferred into injection vials for quantification of short-chain fatty acids (SCFAs) using instrumental analysis.

### 2.9. Statistical Analysis

Growth performance, immune organ indices, immune markers, antioxidant parameters, and morphological data are presented as mean ± SEM. The normality of all data was confirmed, and statistical analyses were conducted using SPSS version 26.0 (IBM Corp., Armonk, NY, USA). Subsequently, one-way ANOVA was performed, followed by Tukey’s post hoc test to determine significant differences among groups. Differences were deemed statistically significant at *p* < 0.05. GraphPad Prism 9.0 (GraphPad Software Inc., San Diego, CA, USA) was employed for figure generation. Data analysis and visualization were performed using BMKCloud. The raw experimental data generated in this study were deposited in the China National GeneBank Sequence Archive (CNSA: https://db.cngb.org/cnsa/[M20.1] (accessed on 6 June 2025)) of the China National Center for Bioinformation under accession number CNP0007514 (submission ID: sub072597).

## 3. Results

### 3.1. Effect of YPF-P on Growth Performance of Goslings

The impact of YPF-P on gosling growth performance is presented in Table 2. Compared with the Control group, all experimental groups significantly decreased the feed to F/G during d1 to 14 (*p* < 0.05), and the YPFPII groups significantly reduced the final body weight at d 21 (*p* < 0.05).

### 3.2. Effects of YPF-P on Serum Antioxidant Indexes

The impact of YPF-P on serum antioxidant capacity in goslings is presented in Table 3. Relative to the Control group, the YPF-PII and YPF-PIII groups showed significant enhancements in the activities of GSH-Px and SOD, along with a significant decrease in MDA levels (*p* < 0.05). Although T-AOC and CAT levels showed an increasing trend in the YPF-*p* groups, the differences were not statistically significant (*p* > 0.05).

### 3.3. Effect of YPF-P on Immunoglobulin Concentrations in Goslings

The impact of YPF-P on the immune organ indices in goslings is presented in Table 4. The results demonstrated that the thymus index and bursa of Fabricius index were significantly higher in the YPFPII and YPFPIII groups relative to the Control group (*p* < 0.05). Although the spleen index tended to increase in all YPF-P treatment groups, the differences were not statistically significant relative to the Control group (*p* > 0.05).

**Figure 1:**
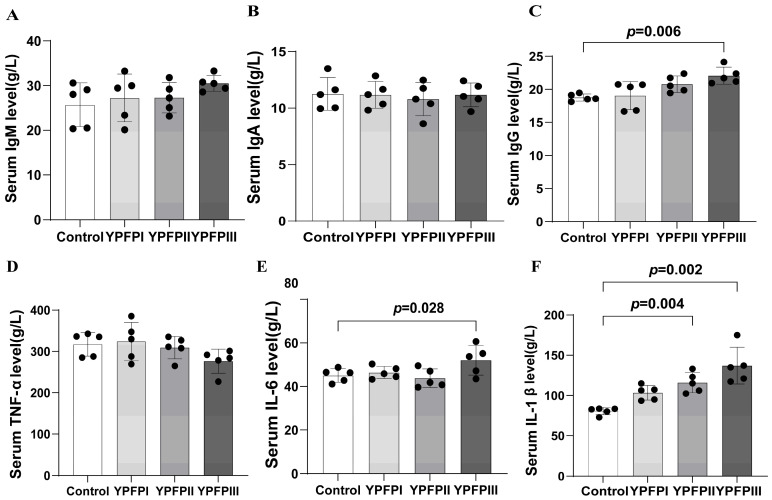
Effects of YPF-P treatment on immune level: (**A**) IgM, (**B**) IgA, (**C**) IgG, (**D**) TNF-α, (**E**) IL-6, and (**F**) IL-1β. Bars with different letters differ significantly (*p* < 0.05). Black dots represent individual sample sizes within each group (n = 5).

The impact of YPF-P on plasma immune parameters in goslings is presented in Figure 1. Relative to the Control group, plasma IgG levels were significantly higher in the YPFPI, YPFPII, and YPFPIII groups (*p* < 0.05; Figure 1C). IL-1β levels increased significantly in the YPFPII and YPFPIII groups (*p* < 0.05; Figure 1F), while IL-6 levels showed significant elevation only in the YPFPIII group (*p* < 0.05; Figure 1E).

### 3.4. Influence of YPF-P on Duodenum, Jejunum, and Ileum Intestinal Morphology

**Figure 2:**
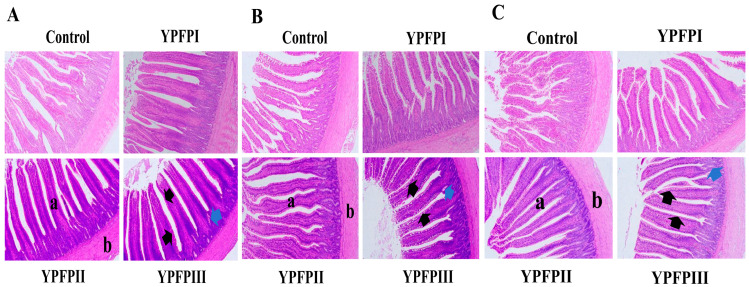
The histomorphological observation of (**A**) duodenum, (**B**) jejunum, and (**C**) ileum in goslings. HE, 100×: (a) intestinal villi, (b) muscle layer; black arrows indicate goblet cells (white vesicular structures) and blue arrows indicate intestinal crypts.

Relative to the Control group, supplementation with 400 mg/kg and 600 mg/kg of YPF-P significantly enhanced villus height (*p* < 0.05; Table 5) and the villus height to crypt depth ratio (V/C) (*p* < 0.05; Table 5). All treatment groups had higher jejunal villus height/crypt depth (V/C) ratios than the Control group, but these differences were not significant (*p* > 0.05; Table 5). YPF-P supplementation improved intestinal morphology across all sections, characterized by elongated and broadened villi, shallower crypts, and increased goblet cell counts (Figure 2A–C).

### 3.5. Influence of YPF-P on Cecal Microflora

**Figure 3:**
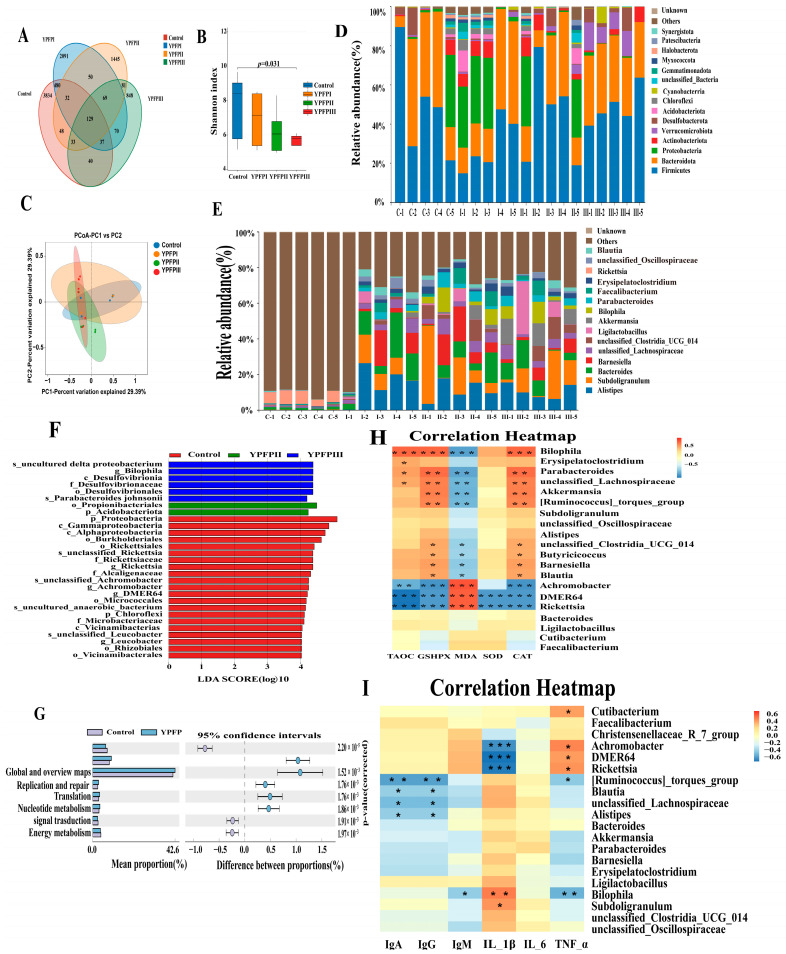
Impacts of YPF-P on the cecal microbiota of goslings: (**A**) Venn analysis diagram: In the above figure, different colors of ellipses represent different samples (or groups). The numbers in overlapping areas indicate the number of species shared among groups, while the non-overlapping areas represent species unique to each group. (**B**) Alpha diversity of the cecal microbiota across different groups was evaluated. Data were analyzed using the nonparametric Kruskal–Wallis test. (**C**) PCoA of cecal microbiota in different groups. (**D**) Relative abundance at the phylum levels of cecal microbiota in goslings (top 10), C-1–C-5: Control; I-1–I-5: YPFPI; II-1–II-5: YPFPII; and III-1–III-5: YPFPIII. (**E**) Relative abundance at the genus levels of cecal microbiota in goslings (top 10), C-1–C-5: Control; I-1–I-5: YPFPI; II-1–II-5: YPFPII; and III-1–III-5: YPFPIII. (**F**) LEfSe analysis of cecal microbiota (LDA score is greater than 2). (**G**) Functional predictive analysis: The above figure shows differential analysis of KEGG metabolic pathways at the second hierarchical level. Different colors represent different groups. The vertical axis on the left side of the figure indicates the abundance ratio of different functions between the two groups. The middle area shows the difference ratio of function abundance within a 95% confidence interval. The value on the far right represents the *p*-value. (**H**) Pearson correlation analysis between intestinal flora and antioxidant substances. (**I**) Pearson correlation analysis between gut microbiota and immune factors. Note * means *p* < 0.05, ** means *p* < 0.01, and *** means *p* < 0.001.

To assess the impact of YPF-P on the intestinal microbiota of goslings, cecal contents were subjected to 16S rRNA gene sequencing. As shown in the Venn diagram (Figure 3), a total of 9387 OTUs were detected in the cecal contents across all treatment groups, with 129 OTUs commonly shared among them. The Control, YPFPI, YPFPII, and YPFPIII groups contained 4733, 2958, 1887, and 1307 unique OTUs, respectively (Figure 3A). The Shannon index was significantly lower in the PFPIII group than in the Control (*p* < 0.05; Figure 3B), while β-diversity showed no significant group differences (Figure 3C). 16S rRNA sequencing revealed that the cecal microbiota at the phylum level was primarily composed of *Firmicutes*, *Bacteroidota*, *Proteobacteria*, and *Actinobacteriota*. YPF-P supplementation led to a shift in the cecal microbial composition, notably increasing the relative abundance of *Firmicutes* and *Bacteroidota*, while reducing Proteobacteria levels, especially in the YPFPIII group (Figure 3D).

At the genus level (Figure 3E), *Alistipes*, *Subdoligranulum*, *Bacteroides*, and *Barnesiella* were predominant. Their relative abundances increased following YPF-P treatment, with *Bacteroides* enriched in YPFPI, *Subdoligranulum* in YPFPII, and *Alistipes* in YPFPIII. Notably, the relative abundance of *Akkermansia* significantly increased from 0.72% in the Control group to 7.58% in the YPFPIII group (*p* < 0.05). LEfSe analysis revealed no significant microbial variations in the YPF-P I group (Figure 3F). In contrast, the Control group had eight distinct taxa, dominated by *Proteobacteria*. YPFPII showed enrichment in *Ruminococcaceae* and *Barnesiella*, while YPFPIII exhibited eleven differentially abundant taxa, including *Alistipes*, *Akkermansia*, and various members of *Verrucomicrobiota* and *Lachnospiraceae*. At the genus level, *Akkermansia* and *Alistipes* were significantly more abundant in YPFPIII than in all other groups (*p* < 0.05), whereas *Rickettsia* was significantly reduced (*p* < 0.05; Table 6). Microbial function prediction via Picrust2 (Figure 3G) indicated that genes involved in carbohydrate and nucleotide metabolism, as well as replication, repair, and translation, were significantly upregulated in the YPF-P groups (*p* < 0.01).

Correlation analysis (Figure 3H) showed that *Akkermansia* was positively associated with antioxidant enzymes (GSH-PX, CAT) and negatively with MDA levels (*p* < 0.01). Other beneficial genera (*Barnesiella*, *Blautia*, unclassified *Lachnospiraceae*, etc.) also showed similar patterns. In contrast, *Rickettsia*, which was enriched in the Control and YPFPI groups, showed significant negative correlations with antioxidant capacity, including T-AOC, GSH-PX, SOD, and CAT, while exhibiting positive correlations with MDA (*p* < 0.001). *Rickettsia*, *DMER64*, and *Achromobacter* negatively correlated with IL-1β, while *Bilophila* was positively correlated (*p* < 0.01 or *p* < 0.001; Figure 3I).

### 3.6. The Effect of Yupingfeng Polysaccharides on Short-Chain Fatty Acids in the Cecum of Goslings

**Figure 4:**
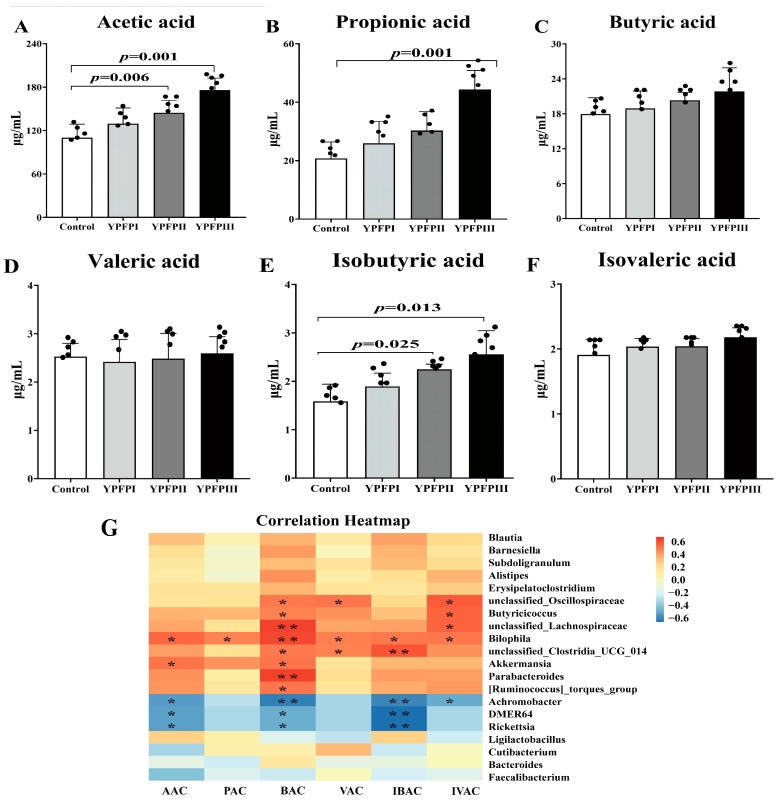
Effects of YPFP on SCFAs in cecum contents of goslings: (**A**) acetic acid, (**B**) propionic acid, (**C**) butyric acid, (**D**) valeric acid, (**E**) isobutyric acid, and (**F**) isovaleric acid. Black dots represent individual sample sizes within each group (n = 5). (**G**) Pearson correlation analysis between intestinal flora and short-chain fatty acids: (AAC) acetic acid, (PAC) propionic acid, (BAC) butyric acid, (VAC) valeric acid, (IBAC) isobutyric acid, and (IVAC) isovaleric acid. Black dots represent individual sample sizes within each group (n = 5). * indicates *p* < 0.05, ** indicates *p* < 0.01.

YPF-P supplementation modified the short-chain fatty acid (SCFA) composition in the cecal contents of goslings. Acetic acid, propionic acid, and butyric acid were the predominant SCFAs. Relative to the Control group, the YPFPII and YPFPIII groups exhibited significantly increased concentrations of acetic acid and isobutyric acid (Figure 4A,E; *p* < 0.05). Additionally, the level of propionic acid was significantly elevated in the YPFPIII group, reaching 44.39 μg/mL, which is approximately 2.14 times higher than that in the Control group (Figure 4B).

YPFP modulated SCFA levels in association with specific gut microbial taxa (Figure 4G). Pearson correlation analysis indicated that elevated SCFA concentrations were positively associated with the enrichment of *Akkermansia*, *Butyricicoccus*, unclassified *Lachnospiraceae*, unclassified *Clostridia UCG-014*, and *Ruminococcus* torques group (*p* < 0.05), while *Rickettsia* abundance was negatively correlated with several SCFAs. Notably, the *Akkermansia* and *Ruminococcus* torques groups were enriched in the YPFPIII and YPFPII groups, respectively, suggesting their potential role in SCFA production.

## 4. Discussion

YPF-P is a complex polysaccharide extracted from *Astragalus*, *Atractylodes macrocephala*, and *Saposhnikovia divaricata*, which is the main active ingredient of Yupingfeng San. Compared with the individually extracted *Astragalus*, *Atractylodes macrocephalae*, and *Saposhnikovia divaricata* polysaccharides, YPF-P exhibits a broader and synergistic enhancement in immunomodulation. Although these single polysaccharides possess various biological activities such as immune-enhancing [16,17,18], antioxidant [16,19,20], anti-inflammatory [21,22] and growth-promoting activities [23,24,25], their effects are relatively limited. On the other hand, YPF-P can not only significantly enhance cellular, humoral, and erythrocyte immunity but also effectively alleviate immunosuppression and protect spleen function, as well as attenuate hepatic injury and pulmonary fibrosis, with a clear dose-dependence and good adaptability [25,26,27]. In addition, YPF-P, as one of the plant polysaccharides, is an alternative to antibiotic feed additives and has been widely used in the field of animal husbandry. Studies have confirmed its significant role in improving animal performance, especially in the growth and development of poultry. Research by Li et al. [28] indicated that diets containing varying levels of *Astragalus* polysaccharides showed a trend of increasing average daily gain and decreasing FCR in broiler chickens aged 1 to 14 days. Similarly, Long et al. [29] found that adding 1 g/kg of *Acanthopanax* polysaccharides to feed significantly increased the average daily gain and average daily feed intake in broilers aged 22 to 42 days, while FCR was significantly reduced. Our results showed that all dosage groups significantly reduced the feed-to-gain ratio of goslings from day 1 to 14. Additionally, adding 400 mg/kg and 600 mg/kg of YPF-P significantly reduced the final body weight of goslings at 21 days of age, which is consistent with the findings of Zhou et al. [30], where adding 1600 mg/kg of *Atractylodes* polysaccharides to the diet reduced the final body weight of goslings aged 1 to 28 days. Therefore, supplementation with an appropriate concentration of YPF-P in the basal diet can reduce the feed-to-gain ratio and improve the growth performance of goslings.

The thymus, bursa of Fabricius, and spleen are the primary lymphoid organs in poultry, serving as key regulatory centers of the immune system [31]. The augmented relative mass of immune organs serves as an indicator of enhanced immunocompetence, implying a potential improvement in immune regulatory efficiency [32]. In our experiment, adding 400 mg/kg and 600 mg/kg of YPF-P significantly increased the thymus and bursa indices, suggesting enhanced immune factor production. Immune organs and plasma immune factors are key indicators of poultry immune function, and plasma IgA, IgG, and IgM levels are leading indicators of humoral immune status. IgG levels were significantly higher in all experimental groups compared with the Control group. This is similar to the findings of Wu et al. [33], who reported that polysaccharides increased plasma IgM, IgG, and IgA levels in 21-day-old broilers fed from day 7. In this study, dietary supplementation with 600 mg/kg of YPF-P significantly increased plasma IL-6 and IL-1β levels in 21-day-old goslings, suggesting an enhanced innate immune response. This is similar to the findings of Long et al. [29] and Kong et al. [34]. In conclusion, the addition of YPF-P to the diet helps improve immune regulation, enhancing infection resistance.

Reactive oxygen and nitrogen species (ROS/RNS) modulate poultry performance, immunity, and product quality by interfering with antioxidant enzyme activity. While moderate ROS/RNS exposure elicits beneficial adaptive responses, excessive accumulation triggers oxidative stress, disrupting cellular redox homeostasis. The activity of antioxidant enzymes serves as a critical biomarker for evaluating oxidative status in poultry [35,36,37]. MDA is a terminal product of lipid peroxidation in plasma. It is widely recognized as a biomarker for oxidative stress. Meanwhile, T-AOC and GSH-Px activity are typically utilized as primary markers to assess serum antioxidant capacity [38]. Our study showed that adding YPF-P significantly increased the activities of GSH-PX and SOD in goslings at 21 days of age, while significantly reducing MDA content. These results are consistent with the findings of Jin et al. [39] and Zhao et al. [40]. Other research has shown that sage polysaccharides were also found to reduce the MDA and H_2_O_2_ activities in a florfenicol-induced liver stress model in chicks, alleviating liver damage caused by florfenicol. In addition, Huang et al. [41] demonstrated that Morinda officinalis polysaccharides could reduce oxidative damage caused by tibial dyschondroplasia in broilers, restoring meat metabolite levels and improving meat quality. In summary, YPF-P can enhance the antioxidant capacity of goslings at 21 days of age and reduce oxidative damage.

Intestinal mucosal epithelial cells are the main cell type that forms the intestinal mucosa, and together with the mucus covering their surface, they constitute the mechanical barrier of the intestine [42]. The structure of intestinal villi is prone to damage from adverse factors, and morphological changes can impair nutrient absorption and the filtering of harmful substances, leading to compromised barrier function. Qiao et al. [43] found that adding astragalus polysaccharides and licorice polysaccharides to the diet significantly improved the V/C ratio in the duodenum, jejunum, and ileum of broiler chickens. These results are consistent with our findings, where adding YPF-P to the diet significantly increased the VH and V/C ratio in the duodenum and ileum of goslings at 21 days of age. These results are consistent with the findings of Zheng et al. [12] and Chen et al. [44]. These findings suggest that YPF-P may exert its effects via improvements in intestinal morphology. The normal gastrointestinal microbiota plays a crucial role in the growth and development of organisms by influencing the morphological development of intestinal villi and crypts, as well as nutrient absorption [45]. Therefore, we conducted a further analysis of the intestinal microbiota.

The relationship between gut microbiota and the host is symbiotic, and the composition of poultry diets can affect the structure of the intestinal microbiota, altering metabolic pathways and metabolites, ultimately impacting host health and production performance [12]. Dietary manipulation of gut microbiota can promote poultry production performance to some extent [46]. Studies suggest that plant polysaccharides, as dietary supplements, improve gut microbiota by promoting the proliferation of beneficial bacteria, inhibiting harmful bacteria, and regulating metabolite levels, thereby enhancing host health [47]. Our study results showed that YPF-P mainly increased the relative abundance of *Akkermansia* and *Alistipes* in the cecum of goslings while decreasing the relative abundance of *Rickettsia*. Both *Akkermansia* and *Alistipes* are closely related to carbohydrate metabolism and are essential for maintaining gut health in poultry, while *Rickettsia*, a pathogen in the phylum *Proteobacteria*, is a common disease-causing agent. *Akkermansia* may play a crucial role in energy absorption, consumption, and fat browning in the host’s gut. Studies have found that *Akkermansia* could reduce diet-induced weight gain and obesity in mice by controlling carbohydrate metabolism and consuming body energy [48,49]. It also improved Salmonella-induced damage to the intestinal mucosa by promoting the proliferation of intestinal epithelial cells, playing a critical role in protecting the intestinal mucosal barrier. *Alistipes* may have immune-regulating effects in cancer [50], but its functions vary with dietary and environmental changes. *Akkermansia* and *Alistipes* are negatively correlated with obesity, and both may be associated with metabolic diseases like insulin resistance, metabolic syndrome, and type 2 diabetes, helping the body consume pro-inflammatory and lipid-accumulating monosaccharides [51]. Studies have shown that medium-dose tea polysaccharides can significantly promote the proliferation of beneficial bacteria such as *Akkermansia* and *Alistipes* in obese mice [52], which is consistent with our findings, suggesting that the decrease in gosling weight may be related to the cecal microbiota, particularly *Akkermansia* and *Alistipes*.

The symbiotic relationship formed between gut microbiota and the host is crucial for maintaining gut homeostasis. Gut microbiota colonization provides a suitable environment for growth, and the co-metabolites produced by the microbiota and the host play essential roles in immune function and growth [53]. Therefore, understanding the structure of gut microbiota in poultry and analyzing its functional predictions can help regulate gut microbes, promoting gut health and growth. Metabolomics can be used to study the metabolic products in the gut, exploring the relationship between microbiota composition and metabolite levels. In serum–microbiome and metabolomics–microbiome correlation analyses, GSH-PX, CAT, acetic acid, and propionic acid showed positive correlations with *Akkermansia*, while triglycerides, uric acid, and MDA were negatively correlated with *Akkermansia*. T-AOC, GSH-PX, SOD, CAT, acetic acid, propionic acid, and isobutyric acid were negatively correlated with *Rickettsia*. Although no significant correlations were found between *Alistipes* and serum/metabolomics indicators, trends were similar to *Akkermansia*, possibly due to joint regulation by other microbiota. These results suggest that the increased relative abundance of beneficial bacteria *Akkermansia* and *Alistipes* may be factors contributing to the increased levels of GSH-PX, CAT, acetic acid, and propionic acid, thereby promoting serum antioxidant capacity, lipid metabolism, and intestinal mucosal barrier function in goslings.

Our results showed that the main upregulated metabolic pathways in the cecum of the goslings in the YPF-P group included genes related to carbohydrate metabolism and nucleotide metabolism, as well as genes involved in “replication and repair” under genetic information processing. This finding suggests that the proliferation of beneficial bacteria induced by YPF-P may enhance the body’s metabolic capabilities. It also demonstrates that beneficial gut bacteria can utilize polysaccharides, breaking them down into nutrients that the host can absorb, thereby promoting host health [54]. However, the exact mechanisms by which plant polysaccharides regulate microbiota structure remain unclear, as do the processes of polysaccharide degradation, transport, and absorption by the body. Future research should focus on a comprehensive exploration of the interactions between polysaccharides, the host, and microorganisms at both the macro and micro levels.

## 5. Conclusions

The findings of this study indicated that dietary supplementation with 400 mg/kg and 600 mg/kg of YPF-P significantly enhanced the growth performance of goslings between days 1 and 14, with the most pronounced effect observed at 600 mg/kg. YPF-P not only reduced the feed conversion ratio but also increased immune organ indices and serum immunoglobulin levels, enhancing antioxidant capacity and immune regulation. Additionally, YPF-P helped maintain the structural integrity of the small intestine and increased the relative abundance of beneficial bacteria such as *Akkermansia*, while reducing harmful bacteria like *Rickettsia*. It also promoted the production of short-chain fatty acids, regulated intestinal microbial balance, and improved gut health. Although no positive Control group was included in this study, the results clearly indicate the significant role of YPF-P in modulating gut microbiota and immune function. To further strengthen the reliability and practical relevance of the findings, future studies should consider including a commonly used herbal antibiotic substitute as a positive control to more comprehensively evaluate the relative efficacy of YPF-P and further validate its potential as an alternative to conventional treatments.

## Figures and Tables

**Table 1:** Composition and nutrient levels of the basal diet.

Material	Content (%)	Nutrient Levels ^(2)^	Content
Corn	56.85	ME (MJ/kg)	11.50
Soybean meal	22.00	CP (g/kg)	17.84
Wheat middling and red dog	10.00	CF (g/kg)	4.61
Rice mill by-product	4.00	Ca (g/kg)	1.00
Limestone	1.50	AP (g/kg)	0.40
Expanded soybean	3.00	Lys (%)	0.89
NaCl	0.30	Met (%)	0.42
CaHPO_4_	1.20	Met + Cys (%)	0.75
DL-Met	0.15		
Premix ^(1)^	1.00		
Total	100.00		

^(1)^ Per kg of feed premix content: Vitamin A 12,000 IU, Vitamin D3 2500 IU, Vitamin E 21.5 mg, Vitamin K3 4.5 mg, Vitamin B1 2.0 mg, Vitamin B2 7.5 mg, Vitamin B6 5 mg, Vitamin B12 20 mg, Niacin 55 mg, Biotin 0.134 mg, Choline 1000 mg, Pantothenic acid 15 mg, Folic acid 1.5 mg, Iron 95 mg, Copper 6 mg, Manganese 100 mg, Zinc 80 mg, Iodine 0.6 mg, Cobalt 0.5 mg, and Selenium 0.4 mg. ^(2)^ Nutrient levels are calculated values. ME, metabolizable energy; CP, crude protein and CF, crude fiber.

**Table 2:** The effect of YPF-P on the production performance of goslings.

Items	Days of Age	Group			
		Control	YPFPI	YPFPII	YPFPIII
IW/g	1	107.45 ± 4.78	107.20 ± 4.80	107.50 ± 3.84	107.15 ± 5.04
FW/g	7	231.77 ± 9.23	231.77 ± 9.23	224.93 ± 4.42	229.98 ± 10.36
	14	577.85 ± 25.41	580.68 ± 24.02	577.91 ± 22.72	574.82 ± 18.27
	21	1015.10 ± 10.57 ^a^	1009.36 ± 12.28 ^a^	987.35 ± 6.67 ^b^	994.16 ± 13.10 ^ab^
ADFI/g	1~7	26.92 ± 1.63	25.99 ± 1.42	25.63 ± 1.57	26.24 ± 2.14
	1~14	57.81 ± 2.98	55.96 ± 2.62	54.85 ± 1.65	55.43 ± 2.11
	1~21	77.67 ± 4.26	76.53 ± 1.18	75.24 ± 1.69	76.55 ± 3.04
ADG/g	1~7	17.76 ± 1.85	17.89 ± 1.24	16.61 ± 1.04	17.55 ± 1.76
	1~14	33.60 ± 2.08	33.81 ± 1.54	33.60 ± 1.58	33.41 ± 1.50
	1~21	43.31 ± 0.81	42.86 ± 0.58	41.98 ± 0.43	42.22 ± 0.87
F/G	1~7	1.52 ± 0.09	1.46 ± 0.09	1.55 ± 0.08	1.50 ± 0.10
	1~14	1.72 ± 0.03 ^a^	1.66 ± 0.05 ^b^	1.63 ± 0.04 ^b^	1.66 ± 0.05 ^b^
	1~21	1.79 ± 0.07	1.79 ± 0.04	1.79 ± 0.05	1.81 ± 0.04

Data are presented as mean ± SEM (n = 60). Different lowercase letters above the bars indicate statistically significant differences (*p* < 0.05), while the absence of letters or shared letters denotes no significant difference (*p* > 0.05). IW, initial weight; FW, weight at the end of the trial; ADFI, average daily feed intake; ADG, average daily gain; and F/G, feed to weight gain ratio. Values are presented as mean ± SEM. Different superscript letters within the same row indicate significant differences (*p* < 0.05).

**Table 3:** Effects of different doses of YPF-P on the serum antioxidant capacity of goslings.

Items	Group			
	Control	YPFPI	YPFPII	YPFPIII
T-AOC/(mM)	0.19 ± 0.01	0.21 ± 0.01	0.22 ± 0.01	0.22 ± 0.02
GSH-PX/(μmol/L)	345.11 ± 70.31 ^c^	364.26 ± 24.32 ^bc^	454.75 ± 21.37 ^b^	565.26 ± 74.16 ^a^
MDA/(nmol/mL)	2.32 ± 0.08 ^a^	2.10 ± 0.23 ^ac^	1.81 ± 0.26 ^bc^	1.70 ± 0.12 ^b^
SOD/(U/mL)	18.24 ± 8.57 ^b^	23.51 ± 2.45 ^ab^	34.03 ± 4.05 ^a^	27.23 ± 6.49 ^a^
CAT/(U/mL)	1.29 ± 0.67	1.43 ± 0.61	1.54 ± 0.36	1.76 ± 0.32

Data are presented as mean ± SEM (n = 5). Different lowercase letters above the bars indicate statistically significant differences (*p* < 0.05), while the absence of letters or shared letters denotes no significant difference (*p* > 0.05). MDA, malondialdehyde; SOD, superoxide dismutase; T-AOC, total antioxidant capacity; and GSH-PX, glutathione peroxidase.

**Table 4:** Effects of different doses of YPF-P on the immune organ index of goslings.

Items	Group			
	Control	YPFPI	YPFPII	YPFPIII
Thymus	2.21 ± 0.46 ^c^	2.29 ± 0.31 ^bc^	2.89 ± 0.45 ^ab^	2.92 ± 0.27 ^a^
Bursa	0.78 ± 0.04 ^b^	0.76 ± 0.05 ^b^	0.92 ± 0.07 ^a^	0.99 ± 0.11 ^a^
Spleen	1.61 ± 0.32	1.69 ± 0.23	1.76 ± 0.35	1.94 ± 0.34

Data are presented as mean ± SEM (n = 5). Different lowercase letters above the bars indicate statistically significant differences (*p* < 0.05), while the absence of letters or shared letters denotes no significant difference (*p* > 0.05).

**Table 5:** Impact of YPF-P on villus height, crypt depth, and VH/CD ratio in goslings.

Items		Group			
		Control	YPFPI	YPFPII	YPFPIII
Villus Height (μm)	Duodenum	665.54 ± 49.80 ^b^	698.02 ± 118.56 ^b^	889.00 ± 130.22 ^a^	968.88 ± 218.31 ^a^
	Jejunum	865.59 ± 154.02	1027.83 ± 149.96	964.56 ± 165.57	941.47 ± 186.76
	Ileum	658.46 ± 142.70 ^c^	755.02 ± 113.68 ^bc^	817.32 ± 146.53 ^ab^	985.70 ± 194.25 ^a^
Crypt Depth (μm)	Duodenum	293.01 ± 35.05	218.55 ± 42.88	229.28 ± 21.26	266.78 ± 73.01
	Jejunum	197.24 ± 44.66	172.06 ± 41.15	221.48 ± 73.53	169.45 ± 40.02
	Ileum	203.16 ± 64.15	184.20 ± 56.36	175.06 ± 45.41	204.60 ± 58.00
V/C ratio	Duodenum	2.30 ± 0.33 ^b^	3.40 ± 1.16 ^ab^	3.90 ± 0.63 ^a^	3.96 ± 1.55 ^a^
	Jejunum	4.69 ± 1.63	6.32 ± 1.94	5.11 ± 2.72	5.89 ± 1.99
	Ileum	3.49 ± 1.20 ^c^	4.44 ± 1.36 ^bc^	5.15 ± 2.32 ^ab^	5.93 ± 1.42 ^a^

Data are presented as mean ± SEM (n = 5). Different lowercase letters above the bars indicate statistically significant differences (*p* < 0.05), while the absence of letters or shared letters denotes no significant difference (*p* > 0.05).

**Table 6:** An abundance of distinct species in each group.

Group	Control	YPFPI	YPFPII	YPFPIII
*Akkermansia* (%)	0.36	0.82	0.72	7.58
*Alistipes* (%)	5.64	8.86	4.42	11.56
*Rickettsia* (%)	4.34	3.74	0.00	0.00

Note: Indicates significant difference compared with the Control (*p* < 0.05).

## Data Availability

The data presented in this study are available upon request from the corresponding author.
